# Mitigating antibiotics misuse in dairy farming systems and milk value chain market: Insights into practices, factors, and farmers education in Nyabihu district, Rwanda

**DOI:** 10.1016/j.onehlt.2024.100843

**Published:** 2024-06-20

**Authors:** Blaise Iraguha, Jean Pierre M. Mpatswenumugabo, Methode Ngabo Gasana, Elina Åsbjer

**Affiliations:** a Heifer International Rwanda, Rwanda Dairy Development Project (RDDP), Rwanda; b University of Rwanda/College of Agriculture, Animal Sciences and Veterinary Medicine, Rwanda; c Department of Clinical Sciences, Swedish University of Agricultural Sciences (SLU), Sweden; d Rwanda Agriculture and Animal Resources Development Board, Rwanda; e Department of Applied Animal Science and Welfare, Swedish University of Agricultural Sciences (SLU), Sweden

**Keywords:** Antibiotics, Antibiotic residues, Antimicrobial resistance, Milk, Milk collection center

## Abstract

The widespread misuse of antibiotics to combat bacterial infections in dairy farming is a global concern contributing to antimicrobial resistance (AMR). To gain insights within small-scale dairy farming, a study was conducted in Nyabihu district of Rwanda from September 2021 to April 2023 to assess practices and factors associated with antibiotic use, investigate antibiotic residues in cow milk and undertake a comprehensive training program to improve quality milk production. A mixed-methods approach, combining cross-sectional and longitudinal intervention studies, involved 42 regular dairy farmers from both open and zero-grazing systems delivering milk to the Union pour la Promotion des Cooperatives des Eleveurs en Nyabihu (UPROCENYA) milk collection center (MCC). Standardized questionnaires and farm interviews were conducted to assess antibiotic use practices while bulk tank milk samples from the same farmers were collected and tested for antibiotic residues using rapid tests over 16 months (8 months before and 8 months after training).

Out of 451 bulk tank milk samples tested, 27 samples (6%) contained antibiotic residues, primarily tetracyclines (55.3%) and beta-lactams (44.7%). Before farmers training, 5182.75 l of milk were rejected monthly due to antibiotic residues. Following training, milk rejections decreased to 3192.75 l per month, reflecting 38.35% monthly decrease. However, no statistically significant difference was found by independent *t*-test (*t* = 1.441; *p* = 0.173) between milk rejected before and after training. 97.6% of interviewed farmers reported using antibiotics within six months preceding data collection, with 71.4% primarily used for disease treatment, notably targeting tick-borne diseases (34.0%). Alarming practices included administering antibiotics without referring samples for laboratory examination (100%), disregarding withdrawal periods (88.1%) and administering antibiotics without a veterinary doctor's prescription (85.7%). Factors contributing to these practices included limited farmer’s knowledge on antibiotics, easy access to antibiotics in local agro-veterinary shops, and insufficient veterinary services. Antibiotic-laden milk was used to feed calves (38.6%), consumed at home (26.5%), and sold (12.0%).

The observed misuse of antibiotics and disregard for antibiotic withdrawal periods pose significant threats to both milk quality and human health. The authors recommend that dairy farmers prioritize animal health monitoring and implementing biosecurity measures to prevent diseases and thus reduce antibiotic usage. Collaborative efforts among stakeholders are highly recommended to enhance capacity building for dairy farmers and support research initiatives. Furthermore, it is strongly suggested to strengthen regulations on the prudent use of antibiotics within the Rwandan food production system to curb antimicrobial resistance across both animal and human populations.

## 1 Introduction

In recent decades, the livestock industry has seen significant advancements driven by global animal health improvements and technological innovations [1]. Rwanda, with a substantial dairy cow population ranging around 1.5 million [2], relies heavily on milk production, which plays a crucial role in childhood development and dairy farmers' economic well-being [3]. The entire country is dominated by zero-grazing system where farmers often keep 1–3 cows entirely confined and fed within a kraal [4], while open grazing system is practiced in parts of the North-Western highlands and the lowland eastern savannah with farmers often raising between 10 and 20 cows that graze freely on individual or communal lands [5]. Additionally, the milk value chain creates employments for several actors including dairy farmers (dairy cattle owners), milk middlemen who act as intermediaries facilitating transactions between producers and buyers, and milk transporters involved in physical movement of milk from producers to other facilities, retailers, milk collection points, MCCs and processors [3]. However, Rwanda faces challenges in livestock management due to the cow population density exceeding the estimated carrying capacity, particularly considering that 77.2% of the population owns <0.5 ha of land per farmer [6]. Furthermore, small-scale dairy farmers struggle to provide adequate feeds of good quality, leading to poor animal health [7].

Insufficient knowledge on antibiotics and antimicrobial resistance among farmers and animal health professionals [36] and high volume of antibiotic importation and sales pose concerns for the Rwandan dairy sector, contributing to antibiotic misuse [8]. Improper use of antibiotics lead to increased livestock mortalities, substantial economic losses due to treatment failure and reduced productivity in terms of quality and yield [33]. These factors contribute to increased production costs for dairy farmers covering expenses for additional medicines and veterinary services among others [10], and potentially undermining consumers' confidence, leading to reduced sales and market share [[34], [37]]. Antibiotic misuse and resistance in dairy farming are closely linked to the treatment of bovine intramammary infections (mastitis) [35]. As a result of drug resistance, bacterial infections become difficult or impossible to treat [13] increasing the risk of disease spread, severe illness, deaths [14], and potential spread of antibiotic-resistant bacteria in the environment [15]. Moreover, antibiotic residues have also been detected in foods of animal origin [16] and can potentially be transmitted to humans consuming animal-derived food and products [17]. Failure to change the current antibiotic use practices may render newly available and future antibiotics ineffective [13].

Despite limited literature on practices and factors associated with antibiotic use in Rwanda; the widespread and unregulated use of antibiotics for diseases treatment, prevention and growth promotion is a common practice across different livestock species in Africa [[18], [19], [20], [21]]. Moreover, adherence with withdrawal periods to ensure that milk from animals receiving antibiotics is safe for consumption is not commonly respected, potentially leading to the inclusion of milk with high antibiotic residues in the milk supply chain and into the local communities [19,21].

Rwanda has established policies and regulations to ensure that produced milk undergoes quality control including testing for antibiotics through milk collection centers (MCCs) [22,23]. Once antibiotic residues are detected in supplied milk, it is rejected, turning into significant income losses for producers and MCCs, likely contributing to milk producers resorting to the informal milk market where controls are less stringent [22]. Despite the existing policies, there is a lack of data on practices and factors related to antibiotic misuse in dairy cattle across different grazing systems. Such information is crucial to facilitate implementation of appropriate measures to reduce antibiotic residues in the milk value chain, thus improving milk quality and income for smallholder dairy farmers.

Therefore, this study aimed (1) to investigate the practices and factors associated with antibiotic misuse for treatment and prevention of bacterial infections in dairy cows among smallholder farmers and (2) to determine if a training intervention aimed at enhancing dairy farmers' knowledge on quality milk production and animal husbandry reduced volumes of milk rejected due to antibiotic residues at UPROCENYA MCC by comparing milk rejections before and after the training.

## 2 Materials and methods

### 2.1 Study area

**Fig. 1:**
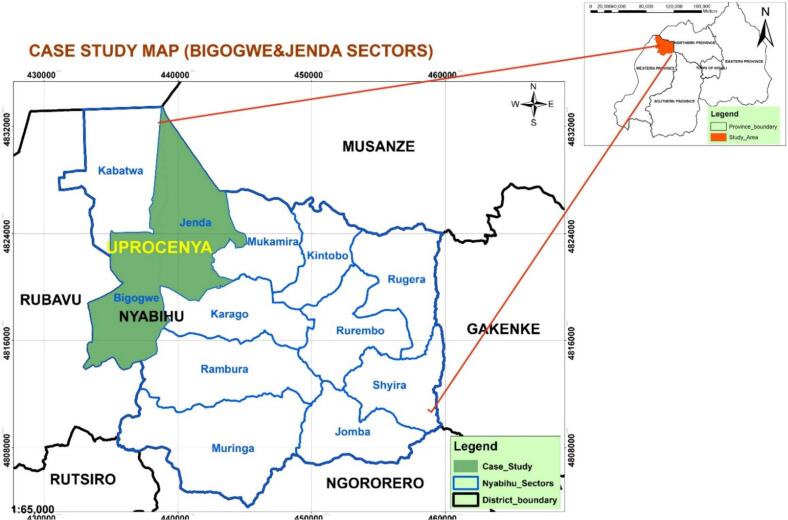
Map of Nyabihu district in Rwanda [25].

This study was carried out in the North-western region of Rwanda, specifically in Nyabihu district Figure 1 (1°39′9.90”S; 29°30′24.62″E) and 2437 m above sea level) [24] during September 2021 to April 2023. Livestock rearing is the prime agricultural activity dominating this area, with 39,628 households, constituting 51.9% of the total district population engaged in livestock farming. These households collectively own approximately 21,600 cows, and the district is home to four active milk collection centers, one large milk processing plant (Mukamira dairy), and nearly 60% of the cheese processors in the country [25].

### 2.2 Target group and sample size

A cross-sectional study was conducted among small-scale dairy farmers supplying milk to the UPROCENYA MCC, chosen for its representation of both cattle grazing systems practiced in Rwanda. While UPROCENYA MCC counts 92 active milk suppliers (52 dairy farmers and 40 milk middlemen), this study targeted dairy farmers only, resulting into 42 dairy farmers selected as regular farmers delivering milk to the MCC consistently throughout the year. Farmers selection also targeted a longitudinal intervention study where the same farmers were followed for a period of 16 months (8 months before the training and 8 months after) to assess antibiotic residues in milk delivered at the MCC. Middlemen were excluded from the study to prevent biases resulting from mixing milk of cows from different farms which could mislead comparison of antibiotic residue test results and assessment of training impact. To ensure a representation of both grazing systems, 42 selected farmers were allocated into two strata: 25 farmers practicing open grazing and 17 practicing zero-grazing.

Stratification allowed to account for variations in antibiotic use practices based on farm size, farm location, management practices, etc., aiming to ensure a diverse representation of the overall population. The selected sample size provided a balance between the need for meaningful insights into the practical constraints and effects of different grazing systems associated with data collection and analysis. With available resources, including time and budget limitations, a sample size of 42 dairy farmers was deemed sufficient while maintaining statistical power.

## 3 Questionnaire design and farmer interviews

Data on practices associated with antibiotic use was collected during farm visit interviews using a standardized questionnaire comprising three parts: socio-demographic information, antibiotic usage practices, and farmers' knowledge on antibiotics (Table 1). The questionnaire included dichotomous (Yes/No), multiple-response questions and open-ended questions to elicit unrestricted written answers. The questionnaire was developed after a review of similar literature and consultation with dairy industry experts to ensure the comprehensiveness and relevance of the study objectives. Prior to data collection, the questionnaire was tested on five farmers to assess its usefulness. The 42 dairy farmers were interviewed at the beginning of the study, and the findings from the interviews served as a basis for subsequent training.

**Table 1:** Summary questionnaire administered to individual dairy farmers.

Variables	Categories
Grazing type	Open or free grazing/ zero grazing
Milk supply location (Milk markets)	MCC/Processor/Kiosks/Informal market
Use of any antibiotic during the last 6 months	Yes/No
Reasons for antibiotics use	Treatment/Prevention/both
Health problems associated with antibiotic use	Mastitis, cough, diarrhea, fever, tickborne diseases, injury/wound/urinary tracts/colic/others
Physical attendance, examination, and sample's analysis to the sick cow by veterinarian before treatment.	Yes/No
Prescription of antibiotics by a qualified veterinarian	Yes/No
Who treated the sick animals/ who administered the medicine by injection	Veterinarian/Myself/cow-keeper/Neighbouring farmer
Respect of withdrawal period as indicated by manufacturer	Yes/No
Respect of frequency of antibiotic administration as indicated by manufacturer	Yes/No
Keeping leftover of antibiotics for future use	Yes/No
Source of antibiotics	From vet pharmacy/shop in community /mobile veterinarian/ MCC/neighbor farmer /others
Availability of private/public veterinarians	Yes/No
Use of milk from animals undergoing antibiotics treatment	Sold/ Drunk/Discarded/Feed calves/Others
Knowledge on antibiotic resistance, effects on animal and human health	Yes/No

### 3.1 Training of farmers

The training took place at UPROCENYA MCC during July and August 2022. Following interview findings, participants were trained using a training manual for smallholder dairy producers from the Food and Agricultural Organization (FAO) (2019) (https://www.fao.org/dairy-production-products/resources/training-material/en/) coupled with Dairy Dynamic Management (DDM) principles emphasizing communication and information sharing [26]. A total of 57 participants (including 11 females) comprising the 42 interviewed farmers and 10 middlemen delivering milk to UPROCENYA MCC attended the training voluntarily. The training covered various topics including milk production best practice through improved animal health, disease control and prevention (e.g. mastitis) and antibiotic use and resistance. Practical sessions were integrated into the training addressing mastitis control through teat dips and subclinical mastitis screening using California Mastitis Test (CMT). All the training sessions were conducted by a dairy expert assisted by MCC technicians.

### 3.2 Antibiotic residues and milk rejections

Over 16 months, a total of 451 bulk milk tank samples (225 before and 226 after the training) were tested using BetaStar® Combo test (Neogen Corporation, Lansing, MI, USA), an immunochromatographic medium that uses a specific receptor attached to gold particles to detect antibiotic residues belonging to the family of tetracyclines and β-lactams. According to the manufacturer's instructions, a milk sample of 200 μL cold milk was homogenized and incubated at room temperature (25 °C ±5), and results were evaluated within 5–7 min by visualizing and examining the intensity of the signal at each test line and compare the intensity to the control line. The test kit was previously used and validated for its sensitivity and specificity compared to Liquid Chromatography Coupled with Tandem Mass Spectrometry (LC-MS/MS) [27].

Bulk tank antibiotic residues data from milk delivered by the 42 farmers was collected daily for eight months before the training (September 2021 to April 2022) and eight months after the training (September 2022 to April 2023). For any discovered antibiotic residues, the entire milk bulk tank was rejected, and the amount recorded. Milk supplied by middlemen and irregular farmers was recorded separately and was not included into the study.

### 3.3 Data management and statistical analysis

Data from the questionnaires were encoded into Excel spreadsheets. Descriptive statistics were used to evaluate the social demographic characteristics and antibiotic use practices. The on-farm antibiotic use practices (independent variables) were correlated with grazing systems using Fisher's exact tests to compare the likelihood of antibiotic use and practices across different grazing systems at a 5% confidence level.

Milk volume rejected due to antibiotic residues was consolidated monthly and used to generate box and whisker graphs for data visualization. Rejected volumes data were checked for normality using the Shapiro-Wilk test, and the means of milk rejected before and after the training were compared using independent *t*-test. All data were imported from Microsoft Excel and analyzed using RStudio.

## 4 Results

### 4.1 Sociodemographic characterization of interviewed respondents

The findings in Table 2 provide an overview of respondent demographics and herd characteristics within different grazing systems. Most respondents were males, and a significant portion were adults aged over 30. On average, a farmer owned 5 cows with significant variations within grazing systems. Additionally, most respondents were married and supplied milk to the MCC. While most respondents possessed basic literacy skills and had completed primary school, their educational attainment was limited.

**Table 2:** Socio-demographic characteristics of participants (*n* = 42).

Variable			Grazing system	
	Category	Total	Open grazing (*n* = 25)	Zero grazing (*n* = 17)
Cow ownership	Mean Cows [IQR]/ Farmer	5 [1−20]	8 [4–20]	2 [1–3]
Milking Cows	Mean Cows [IQR]/ Farmer	3 [1−10]	4 [1–10]	1 [1–2]

		**N (%)**	**N (%)**	**N (%)**
Sex	Male	32 (76.2)	23 (92.0)	9 (52.9)
	Female	10 (23.8)	2 (8.0)	8 (47.1)
Age	Youth (18–30 years)	13 (30.9)	6 (24.0)	7 (41.2)
	Adult (>30 years)	29 (69.0)	19 (76.0)	10 (58.8)
Marital status	Single	10 (23.8)	8 (32.0)	2 (11.8)
	Married	32 (76.2)	17 (68.0)	15 (88.2)
Occupation	Farmer only	40 (95.2)	25 (100)	15 (88.2)
	Other occupations	2 (4.8)	–	2 (11.8)
Educational level	Illiterate	5 (11.9)	3 (12.0)	2 (11.8)
	Read and write	8 (19.0)	4 (16.0)	4 (23.5)
	Primary	16 (38.0)	11 (44.0)	5 (29.4)
	Senior 6- Secondary	7 (16.7)	5 (20.0)	2 (11.8)
	College and above	6 (14.3)	2 (8.0)	4 (23.5)
Milk supply location	MCC	32 (76.2)	21 (84.0)	11 (64.7)
	Processors	7 (16.7)	3 (12.0)	4 (23.5)
	Middleman	3 (7.1)	1 (4.0)	2 (11.8)
Farm location	Rural	42 (100)	25 (100)	17 (100)
Breed	Cross breed	42 (100)	25 (100)	17 (100)

### 4.2 Practices associated with antibiotic use

Out of the 42 interviewed dairy farmers, 97.6% used antibiotics at least once during six months preceding data collection. No significant difference was observed between open and zero grazing systems (*p* > 0.05). Most antibiotics were used for disease treatment, often administered by farmers themselves without veterinary professionals' oversight. Additionally, most respondents treated their animals with antibiotics without veterinary doctors' prescription and referring samples for laboratory examination before treatment. Furthermore, a majority of the farmers did not adhere to withdrawal periods (Table 3).

**Table 3:** Antibiotic use practices and variability within grazing systems (*n* = 42).

	Total responses	Open grazing (n = 25)	Zero grazing (n = 17)	X-square values	*P*-Value
Question/variable (Answer)	N (%)	N (%)	N (%)		
Have you used antibiotics in the last 6 months **(Yes)**	41 (97.6)	25 (100)	16 (94.1)	1.5065	0.407
What was the reasons for antibiotic use					
Treatment	30 (71.4)	18 (72.0)	12 (70.5)	0.009	1
Prevention	12 (28.6)	7 (28.0)	5 (29.5)		
Did a veterinarian examine the animal before treatment? **(No)**	29 (69.0)	18 (72.0)	11 (64.7)	0.251	0.736
Did a veterinarian refer samples for laboratory examination before treatment? **(No)**	42 (100)	25 (100)	17 (100)	1.523	0.254
Did you get veterinary prescription before treatment **(No)**	36 (85.7)	23 (92.0)	13 (76.4)	1.992	0.203
Route of administration					
Intra-muscular	38 (90.5)	23 (92.0)	15 (88.2)	0.166	1
Intra-mammary	4 (9.5)	2 (8.0)	2 (11.8)		
Did you adhere to the frequency of injection (as indicated by manufacturer) **(No)**	33 (78.6)	24 (96.0)	9 (52.9)	13.763	0.009
Did you discontinue the therapy once symptoms subsided **(Yes)**	28 (66.7)	17 (68.0)	11 (64.7)	1.828	0.477
Who treated the animal?					
A veterinarian	8 (19.0)	4 (16.0)	4 (23.5)	0.372	0.709
A farmer	32 (81.0)	21 (84.0)	13 (76.5)		
How frequent did you treat the cow?					
Single injection	16 (38.1)	6 (24.0)	10 (58.8)	5.838	0.056
Twice	7 (16.7)	6 (24.0)	1 (5.8)		
Three times	19 (45.2)	13 (52.0)	6 (35.2)		
Did you respect withdrawal time (as indicated on the bottle)? **(No)**	37 (88.1)	22 (88.0)	15 (88.2)	0.154	1
Do you keep leftover antibiotics for future use? **(Yes)**	28 (66.7)	19 (76.0)	9 (52.9)	2.421	0.186

### 4.3 Knowledge of farmers on antibiotics, origin of antibiotics and access to veterinary services

The respondents exhibited varying levels of familiarity with different antibiotics, with tetracyclines and penicillin being the most recognized and utilized. Most of the participants were unaware of antibiotic resistance. Additionally, a majority of farmers reported feeding to their calves or use for home consumption milk from animals undergoing antibiotic treatment. Most dairy farmers purchased antibiotics from local veterinary shops, and the primary reason for treatment was tick-borne diseases. While private veterinary technicians were acknowledged for interventions, access to public veterinarians was limited (Table 4).

**Table 4:** General knowledge about antibiotics and their sources.

Variable/Question		Total	Open grazing	Zero grazing
	Category	N (%) based on answers	N (%) based on answers	N (%) based on answers
Antibiotics known by farmers⁎	Penicillin	13 (27.7)	9 (29)	4 (25)
	Oxytetracycline	23 (48.9)	17 (54.8)	6 (37.5)
	Biomycin	4 (8.5)	2 (6.4)	2 (12.5)
	Others e.g. Fluoroquinolones	7 (14.9)	3 (10)	4 (25)
		*N* = 47	31	16
Most used antibiotic during the last 6 months⁎	Penicillin	13 (23.2)	9 (30)	4 (15.3)
	Oxytetracycline	26 (46.4)	16 (53.3)	10 (38.4)
	Biomycin	6 (10.7)	2 (6.6)	4 (15.3)
	Fluoroquinolones	11 (19.6)	3 (10)	8 (30.7)
		*N* = 56	30	26
Health problems treated/concerned⁎	Mastitis	6 (12.8)	4 (13.3)	2 (11.7)
	Diarrhea	4 (8.6)	2 (6.6)	2 (11.7)
	Uknown disease	10 (21.3)	8 (26.6)	2 (11.7)
	Tick-borne diseases	16 (34)	7 (23.3)	9 (52.9)
	Others: injuries, lameness	11 (23.4)	9 (30)	2 (11.7)
		N = 47	30	17
Where do you get antibiotics from? ⁎	From small veterinary shops in the communities	42 (89.3)	25 (89.2)	17 (89.5)
	From MCC	5 (10.6)	3 (10.7)	2 (10.5)
		N = 47	28	19
What do you do with milk from animals undergoing antibiotic treatment? ⁎	Sold	10 (12)	4 (8.1)	6 (17.6)
	Consumed at home	22 (26.5)	13 (26.5)	9 (26.4)
	Discarded	5 (6)	3 (6.1)	2 (5.8)
	Feed to calves	32 (38.6)	19 (38.7)	13 (38.2)
	Others (e.g., pigs)	14 (16.8)	10 (20.4)	4 (11.7)
		*N* = 83	49	34
Are private veterinary technicians available at any time needed for veterinary assistance?	Yes	34 (81.0)	19 (76)	15 (88.2)
	No	8 (19.0)	6 (24)	2 (11.8)
		42	25	17
Are public veterinarians available at any time needed for veterinary assistance	Yes	19 (45.2)	10 (40)	9 (53)
	No	23 (54.8)	15 (60)	8 (47)
		42	25	17
What is the level of satisfaction with medical services provided by veterinarians?	Satisfied	19 (45.2)	8 (32)	11 (64.7)
	Unsatisfied	23 (54.8)	17 (68)	6 (35.3)
		42	25	17
Can antibiotic cure all diseases	Yes	17 (40.4)	13 (52)	4 (23.5)
	No	25 (59.6)	12 (48)	13 (76.5)
		42	25	17
Have you heard about antibiotic resistance?	Yes	8 (19.0)	5 (20)	3 (17.6)
	No	34 (81.0)	20 (80)	14 (82.4)
		42	25	17

⁎ Multiple-choice responses.

### 4.4 Antibiotic residues and milk rejections

**Fig. 2:**
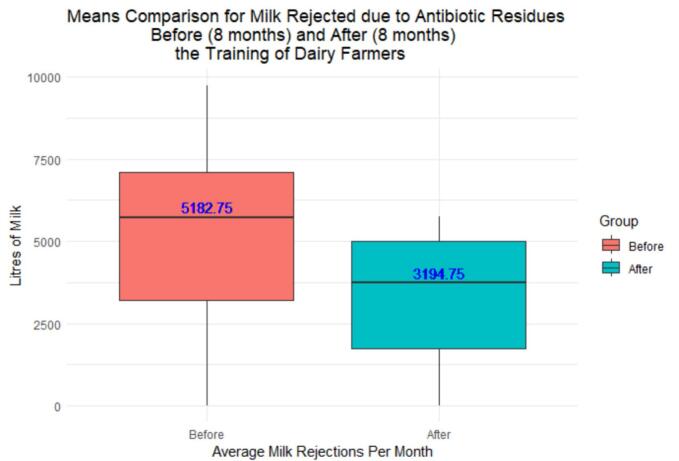
Box and whisker plot of milk rejected monthly at the MCC due to antibiotic residues assessed using rapid tests within 16 months (milk collected from monitored 42 dairy farmers).

Twenty-seven out of 451 bulk milk samples tested positive for antibiotic residues (6%). Out of 27 positive samples, 17 were found over 8 months before the training, decreasing to 10 samples after the training. Within eight months before training, an average of 5182.75 l of milk were rejected monthly due to antibiotic residues, totaling 41,462 l out of 835,543 collected liters (equivalent to 4.9%). After training, the average decreased to 3194.75 l per month, accounting for 25,558 l out of 1,031,127 collected liters (equivalent to 2.5%), reflecting 38.35% decreased monthly rejections. However, no statistically significant difference was found by independent *t*-test (*t* = 1.441; p = 0.173; 95% CI: −993.809, 4969.809) between the amount of milk rejected before and after the training. Residues from tetracyclines accounted for 55.3% of rejected milk while beta-lactams comprised 44.7%. Figure 2

## 5 Discussion

The relationship between antibiotic residues in milk and farmers' practices, as well as factors associated with antibiotic use for treatment and/or prevention of diseases in the Rwandan dairy industry, have not been previously investigated. In the current findings, 97.6% of respondents had used antibiotics in their cattle at least once within the last six months with no statistically significant difference observed between the grazing systems. A high proportion of farmers (69%) in both grazing systems administered antibiotics for animal treatment without a veterinarian's physical examination and 30% used antibiotics for prophylactic purposes rather than treatment. Similar trend was identified in the Eastern Province of Rwanda where the majority of farmers (97.4%) used antibiotics in their farm animals [20]. However, this figure is slightly higher compared to the 71.3% reported in Wakiso, Uganda, emphasizing regional similarities in high antibiotic demand [18]. In this study, tetracyclines emerged as the most used antibiotic class (46.4%) followed by penicillins (23.2%) for dairy cattle treatment. Substantial levels of antimicrobial usage, including tetracyclines, beta-lactams/aminoglycosides, and fluoroquinolones, have been documented across various sub-Saharan African countries [28]. In Nigerian pastoral communities, for instance, high levels of tetracyclines (96.6%), tylosin (95.6%), and penicillin (94%) usage were reported, highlighting the dominant use of tetracyclines and penicillin [29]. The use of broad-spectrum antibiotics for treatment and prevention raises concerns about antibiotic resistance, and more narrow-spectrum antibiotics are recommended [30].

Additionally, 85.7% of the farmers in this study purchased antibiotics without a veterinary doctor prescription. Easy access to antibiotics is noteworthy, with 89.3% of the dairy farmers purchasing antibiotics from local agro-veterinary drug shops, and 10.6% obtaining them from neighbouring MCCs. This accessibility underscores the importance of regulating antibiotic distribution channels to curb potential misuse. Similar findings were reported from studies in Tanzania, Kenya, Ghana, Zambia, and Zimbabwe where increased usage of antibiotics was linked to drug accessibility through privately owned veterinary shops in local settings [19]. In addition, a study in Kenya observed that approximately 40% of the antibiotics were sold in local shops without veterinary prescriptions, with price and customer preference being the determining factors for the type of antibiotics purchased [21]. The unrestricted use of antibiotics among dairy farmers is potentially linked to easy access to antibiotics and limited access to veterinary services. It may also stem from farmers' limited understanding of antibiotic usage and its associated risks, including antibiotic resistance, as shown by this study. In addition, cultural beliefs and habits perpetuate antibiotic misuse, with local farmers often relying on their own judgment and experience. This pattern of attitudes and practices aligns with findings from studie in Tanzania, Kenya, Ghana, Zambia and Zimbabwe, where individuals closely associated with farm animals rely on their own experience and knowledge to make decisions regarding the use of antibiotics, and related practices [19]. Additionally, gaps in regulations and enforcement regarding the use and disposal of veterinary drugs, especially antibiotics, further exacerbate these issues.

The observed non-compliance with antibiotic withdrawal periods (88.1%) poses a significant risk for milk rejection and highlights the need for education of dairy farmers and strict policy enforcement. Similar trends were reported in Kenya and Nigeria [21,29]. In addition, 81% of the respondents had never heard about antibiotic resistance, another knowledge gap requiring urgent attention. Poor knowledge about antibiotic misuse was also reported among Nigerian pastoral communities [29], where about 70.1% of the pastoral communities did not know what misuse of antibiotics entailed. Mastitis has previously been recognized as one of the most important diseases associated with antibiotic use in dairy cattle, while in this study, tick-borne diseases were the main reason for antibiotic treatments. Whether this reflects a true prevalence is unclear and cannot be assessed from this study since many farmers treated their animals themselves without veterinarian's physical examination. Additionally, mastitis could potentially be underdiagnosed or neglected. Further investigations to explore diseases epidemiology and treatment patterns is recommended.

Furthermore, a significant proportion of milk from cows undergoing antibiotic treatment was reported to be fed to calves (38.6%) or consumed at home (26.5%). These practices pose a risk for shedding faecal antibiotic-resistant bacteria into the environment, and hence failure for future antibiotic treatment in both humans and animals [31]. Additionally, the sale of such milk (12%) increases the probability of disseminating antibiotic residues throughout the milk supply chain, potentially contributing to the development of antibiotic resistance. Such public health risk trends have also been documented in various low-income countries [3,21,29]. The current study revealed that 81% of the farmers treated their animals themselves without veterinary examination and prescription. Contributing factors may lie in the associated costs which include veterinary clinical examinations, transport costs, etc. Another contributing factor is Rwanda's challenging topography, characterized by hilly terrain [25], which impedes access to veterinary professionals. This often leads farmers to self-administer treatments without proper oversight.

In this study, 6% of the bulk milk tank samples tested positive for antibiotic residues with tetracyclines accounting for 55.3% and penicillin for 44.7% of the total detections. This finding contrasts with the undetected antibiotic residues in MCC milk samples previously reported in Rwanda [32]. The absence of antibiotic residues in the previous study may be attributed to geographical variations and limited scope of the study, which tested only 32 bulk milk samples, potentially limiting the representativeness of the results. Although the monthly milk rejection due to antibiotic residues declined from 5182.75 l from 8 months before the training to 3194.75 l up to 8 months after the training, the differences before and after lacked statistical significance. The lack of significance is attributed to the substantial variation in monthly milk rejection rates, ranging from 0 to 5000 l per month, indicating the absence of consistent trends. Moreover, milk rejection observations decreased from 17 to 10 bulk tank milk positive samples within 8 months before and after the training, respectively. While there could be confounding factors at play, this declining trend post-intervention suggests that dairy farmers better adhered to withdrawal periods underscoring the positive impact of training interventions to improve milk quality and reducing economic losses at farms and MCCs.

## 6 Conclusion and recommendations

Antibiotic misuse occurs in the study area with no significant difference between open and zero-grazing systems. A substantial knowledge gap regarding appropriate use of antibiotics was identified among the dairy farmers coupled with easy access to antibiotics sold without veterinary doctor's prescriptions and administered by farmers themselves, leading to non-adherence with treatment frequency and non-compliance with antibiotic withdrawal periods. Moreover, dairy cows were often treated without referring to laboratory examination results, and milk from animals undergoing antibiotic treatment was allowed for home consumption. Fortunately, the study indicated positive impact of training interventions on improving quality milk production, reducing milk rejections, and mitigating economic losses incurred by disregarding antibiotic withdrawal periods on farms. It is therefore strongly recommended that dairy farmers monitor their cows' health regularly, ensuring compliance with biosecurity measures and improve dairy farming best practices to reduce the need for and unnecessary use of antibiotics.

. Stakeholders in the dairy value chain should focus their interventions on training programs aimed at reaching more farmers and other actors in the value chain to reduce antibiotic misuse. In addition, engaging veterinary services, academic institutions, private sector, policymakers, and legislative bodies regarding antibiotics sale, prescription, use, and disposal is highly recommended. Lastly, the study calls for further research on antimicrobial resistance (AMR) patterns and public health implications throughout the entire food chain.

## CRediT authorship contribution statement

**Blaise Iraguha:** Conceptualization, Data curation, Formal analysis, Investigation, Methodology, Writing – original draft. **Jean Pierre M. Mpatswenumugabo:** Writing – review & editing. **Methode Ngabo Gasana:** Investigation, Writing – review & editing. **Elina Åsbjer:** Writing – review & editing.

## Declaration of competing interest

The authors declare that they have no known competing financial interests or personal relationships that could have appeared to influence the work reported in this paper.

## Data Availability

Data will be made available on request.

## Appendix A Supplementary data

Supplementary material: insights on antibiotics misuse in Rwandan dairy farming and milk value chains. Supplementary material

## References

[1] Jiang B., Tang W., Cui L., Deng X. (2023). Precision livestock farming research: a global Scientometric review. Animals.

[2] MINAGRI, “RWANDA'S AGRICULTURE SECTOR TRANSFORMATION JOURNEY OVER THE LAST 29 YEARS.” Accessed: May 15, 2024. [Online]. Available: https://www.minagri.gov.rw/updates/news-details/rwandas-agriculture-sector- transformation-journey-over-the-last-29-years.

[3] J. B. Emily ter Steeg, “Investment opportunities in the Rwanda Dairy sector,” Traide. Accessed: May 09, 2024. [Online]. Available: https://static1.squarespace.com/static/633f7112f252f6538fa8ed01/t/63d77e895b9b6910ffdd423f/1675067022314/BORDairy_Rwanda2019_ver4.pdf.

[4] Bishop H., Pfeiffer D. (2008). Factors effecting reproductive performance in Rwandan cattle. Trop. Anim. Health Prod..

[5] Beyi A.F., Dahl E.G. Feed the Future Innovation Lab for Livestock Systems Rwanda: Animal Source Foods Production and Marketing Brief. https://livestocklab.ifas.ufl.edu/media/livestocklabifasufledu/pdf-/pdfs-by-country-pre2019/Rwanda_Brief_ASFProdMkt_final.pdf.

[6] NISR (2021). Agricultural Household Survey 2020 Report. https://statistics.gov.rw/publication/agricultural-household-survey-2020.

[7] De Vries A., Kaylegian K.E., Dahl G.E. (2020). MILK symposium review: improving the productivity, quality, and safety of milk in Rwanda and Nepal. J. Dairy Sci..

[8] Manishimwe R. (2024). Importation trends in antibiotics for veterinary use in Rwanda: a retrospective study between 2019 and 2021. PLoS One.

[10] Chatikobo P. (2010). Mastitis control for quality milk. Dairy Mail Africa.

[13] Prestinaci F., Pezzotti P., Pantosti A. (2015). Antimicrobial resistance: a global multifaceted phenomenon. Pathog. Glob. Health.

[14] Murray C.J. (2022). Global burden of bacterial antimicrobial resistance in 2019: a systematic analysis. Lancet.

[15] Samreen I., Ahmad H.A. Malak, Abulreesh H.H. (2021). Environmental antimicrobial resistance and its drivers: a potential threat to public health. J. Glob. Antimicrob. Resist..

[16] Van T.T.H., Yidana Z., Smooker P.M., Coloe P.J. (2020). Antibiotic use in food animals worldwide, with a focus on Africa: Pluses and minuses. J. Glob. Antimicrob. Resist..

[17] Arsène M.M.J. (2022). The public health issue of antibiotic residues in food and feed: causes, consequences, and potential solutions. Vet. World.

[18] Nayiga S., Kayendeke M., Nabirye C., Willis L.D., Chandler C.I.R., Staedke S.G. (2020). Use of antibiotics to treat humans and animals in Uganda: a cross-sectional survey of households and farmers in rural, urban and peri-urban settings. JAC-Antimicrobial Resist..

[19] Caudell M.A. (2020). Towards a bottom-up understanding of antimicrobial use and resistance on the farm: a knowledge, attitudes, and practices survey across livestock systems in five African countries. PLoS One.

[20] Manishimwe R., Nishimwe K., Ojok L. (2017). Assessment of antibiotic use in farm animals in Rwanda. Trop. Anim. Health Prod..

[21] Kemp S.A., Pinchbeck G.L., Fèvre E.M., Williams N.J. (2021). A cross-sectional survey of the knowledge, attitudes, and practices of antimicrobial users and providers in an area of high-density livestock-human population in Western Kenya. Front. Vet. Sci..

[22] Iteka A., Ministerial M., Arrêté O. (2016).

[23] Habiyaremye N., Ouma E.A., Mtimet N., Obare G.A. (2021). A review of the evolution of dairy policies and regulations in Rwanda and its implications on inputs and services delivery. Front. Vet. Sci..

[24] NISR (2018). Agricultural Household Survey 2017 Report. http://www.statistics.gov.lk/agriculture/Publications/AHS/AHS2016-17Report.pdf.

[25] NISR (2023). The Rwanda 5th Population and Housing Census, District Profile. https://www.statistics.gov.rw/publication/rphc5-district-profile-nyabihu.

[26] Cullor J.S., Doyle M., Garcia S.N., Venkatapuram P., Nandi S. (2015). 54th Annual National Mastitis Council (NMC), Memphis, Tennessee.

[27] Meklati F.R. (2022). Comparative assessment of antibiotic residues using liquid chromatography coupled with tandem mass spectrometry (LC-MS/MS) and a rapid screening test in raw Milk collected from the north-central Algerian dairies. Toxics.

[28] Azabo R., Dulle F., Mshana S.E., Matee M., Kimera S. (2022). Antimicrobial use in cattle and poultry production on occurrence of multidrug resistant Escherichia coli. A systematic review with focus on sub-Saharan Africa. Front. Vet. Sci..

[29] Alhaji N.B., Isola T.O. (2018). Antimicrobial usage by pastoralists in food animals in north-Central Nigeria: the associated socio-cultural drivers for antimicrobials misuse and public health implications. One Heal..

[30] Melander R.J., Zurawski D.V., Melander C. (2018). Narrow-spectrum antibacterial agents. Medchemcomm.

[31] Ricci Antonia (2017). Risk for the development of antimicrobial resistance (AMR) due to feeding of calves with milk containing residues of antibiotics. EFSA J..

[32] Ndahetuye J.B. (2020). MILK symposium review: microbiological quality and safety of milk from farm to milk collection centers in Rwanda. J. Dairy Sci..

[33] Bengtsson B., Greko C. (2014). Antibiotic resistance-consequences for animal health, welfare, and food production,” *Ups*. J. Med. Sci..

[34] Ding Q., Gao J., Ding X., Huang D., Zhao Y., Yang M. (2022). Consumers’ knowledge, attitude, and behavior towards antimicrobial resistance and antimicrobial use in food production in China. Front. Public Heal..

[35] de Jong E. (2023). Invited review: Selective treatment of clinical mastitis in dairy cattle. J. Dairy Sci..

[36] Mugwaneza D. (2024). Factors Associated with Inappropriate Use of Antibiotics Among Animal Health Professionals in Selected Districts of Rwanda, 2021. J. Epidemiol. Glob. Health.

[37] Zhou Y., Zhang A., van Klinken R.D., Wang J. (2024). The effect of information provision on consumers’ risk perceptions of, support for a ban, and behavioral intention towards the preventive use of antibiotics in food animals. BMC Public Health.

