# Supplementary material for: Mitigating antibiotics misuse in dairy farming systems and milk value chain market: Insights into practices, factors, and farmers education in Nyabihu district, Rwanda
Source: One Health. 2024 Jun 20;19:100843. doi: 10.1016/j.onehlt.2024.100843 (PMC11255097; doi:10.1016/j.onehlt.2024.100843)
Supplement: Supplementary file 1 — Supplementary material: insights on antibiotics misuse in Rwandan dairy farming and milk value chains. [file mmc1.docx]

APPENDIX

Questionnaire

# Part I: Socio demographic factors of respondent

Questionnaire ID:

……………………………………………………………..

| **QUESTIONS ON THE FARMER** | | |
| --- | --- | --- |
| **Sr. No** | **Questions** | **Choice Answers** |
| 101 | Residence | District…………………  Sector…………………..  Cell…………………….  Village………………… |
| 102 | Sex | 1. Male 2. Female |
| 103 | Age of the farmer | in years: |
| 104 | Marital status | 1. Single 2. Married 3. Divorced 4. Widowed 5. Separated |
| 105 | What is your occupation? | 1. Farmer only 2. Other regular work (e.g. government, private sector, NGO, etc.) 3. No regular work |
| 106 | Educational level | 1. Illiterate 2. Read and write 3. Primary 4. Senior 6 (secondary) 5. College and above |
| 107 | Average monthly family income | in Rwandan francs |
| 108 | Where do you supply milk | 1. MCC 2. Processor 3. Kiosks 4. Hawkers 5. Others, specify |
| **QUESTIONS ON COWS AND FARM** | | |
| 109 | Farm location | 1. Urban 2. Peri-urban 3. Rural |
| 110 | Number of cows owned | Number …………Milking cows……… |
| 111 | Production system | 1. Extensive 2. Semi-intensive 3. Intensive (zero-grazing): |
| 112 | Breeds | 1. Local 2. Cross breeds 3. Pure exotic breeds |

Part II. Use of antibiotics by the farmers during treatment or prevention

| **Sr. No** | **Item** | **Response** | **Remark** |
| --- | --- | --- | --- |
| 201 | Have you used any antibiotic during the last 6 months? | 1. Yes 2. No |  |
| 202 | For what reason have you used antibiotics? | 1. Treatment 2. Prevention 3. Both |  |
| 203 | Which medicine(s)/antibiotics do you know?  Can you mention its (their) importance? | 1. Penicillin 2. Amoxicillin 3. Gentamicin 4. Ox-tetracycline 5. Chloramphenicol 6. Biomycin (Multiject) 7. Other specify |  |
| 204 | From the above list of antibiotics, which ones did you use in the last 6 months? |  |  |
| 205 | For what health problem have you used antibiotic (s) to cow’s (treatment or prevention)? | 1. Mastitis 2. Cough 3. Diarrhea 4. Fever 5. Tick borne diseases. 6. Injury/Wound 7. Urinary tract symptoms 8. Colic 9. Others, specify | If yes to Q 204 |
| 206 | Did the vet examined the cow before you treated it? | 1. Yes 2. No | If yes to Q 205 |
| 207 | Did the vet took any samples to the laboratory for examination? | 1. Yes 2. No | If yes to Q 205 |
| 208 | Did you get a prescription from the veterinarian? | 1. Yes  2. No |  |
| 209 | Which quantity did you inject at a single injection? | Specify | If yes to Q 204  and 205 |
| 210 | What was the main route of administration for the antibiotic? | 1. Intra-muscular 2. Intra-mammary 3. Subcutaneous 4. Parenteral | If yes to Q 204  and 205 |
| 211 | For how many days did you treat the cow/ inject the medicine? | 1. Single injection 2. Twice 3. Three times 4. Don’t know | If yes to Q 204  and 205 |
| 212 | Did you respect frequency for injection? | 1. Yes 2. No 3. Don’t know |  |
| 213 | Did you discontinue therapy once  the symptoms subsided? | 1. Yes 2. No |  |
| 214 | How often do you use non- prescribed antibiotics? | 1. Never 2. Rarely 3. Often 4. Very often 5. Don’t know |  |
| 215 | Who treated the animal (Injected the medicine) | 1. Veterinarian 2. Myself/My cow-keeper 3. A neighboring farmer |  |
| 216 | What was the medicine withdrawal period? | 1. For milk……….. 2. For meat……….. 3. Don’t know |  |
| 217 | Did you respect withdrawal time? | 1. Yes 2. No 3. Don’t know |  |
| 218 | Do you keep leftovers antibiotics for  future use? | 1. Yes 2. No |  |
| 219 | Use of antibiotics | 1. Inappropriate* 2. Appropriate |  |

Note: The inappropriate use of antibiotic is its misuse through one or more circumstances: use antibiotics without veterinary prescription, not respect withdrawal time, self-medication, do not respect frequency of injection, under dosage or over dosage, treat inappropriate health problem.

# Part III: Origin, knowledge on antibiotics and service offered.

| **Sr. No** | **Questions** | **Choice Answers** |
| --- | --- | --- |
| 301 | Where do you get the antibiotics from? (Multiple response) | 1. From vet pharmacy/shop in community 2. From sector veterinarian 3. From MCC 4. Ambulatory veterinarian 5. Lend from neighbor/farmer 6. Others |
| 302 | How often do you buy medicine at the veterinary pharmacy per year? | 1. Not at all 2. Once 3. Twice 4. Three times 5. Four times 6. >5 times 7. Don’t remember |
| 303 | Are private veterinarians available at any time you seek for them? | 1. Yes 2. No |
| 304 | Are public veterinarians (e.g. sector or disr) available at any time you seek for them? | 1. Yes 2. No |
| 305 | What is the level of satisfaction with the medical services provided by veterinarians? | 1. Satisfied 2. Unsatisfied 3. Don’t know |
| 306 | Which antibiotic do you often use? | 1. Penicillin 2. Amoxicillin 3. Gentamicin 4. Ox-tetracycline 5. Chloramphenicol 6. Other specify------------------------------- |
| 307 | If yes to 305; where did you get information about them? | 1. Relatives 2. Friends 3. Society 4. Veterinarians/physicians 5. Radio/TV (Mass media) 6. Others, specify |
| 308 | Can tetracycline cure all disease? | 1. Yes 2. No 3. Don’t know |
| 309 | Can antibiotics prepared for human be used to  animals? | 1. Yes 2. No 3. Don’t know |
| 310 | What management measures do you take when a cow is milking? |  |
| 311 | In which order do you milk cows undergoing antibiotic treatment is milked? | a) First  b) Between milking  c) Last |
| 312 | What do you do with milk from animals undergoing antibiotics treatment? | a) Sold  b) Drunk  c) Withheld  d) Feed calves  e) Others, specify……………………….. |
| 313 | Have you heard about antibiotic resistance? | 1. Yes 2. No |
| 314 | If yes, what do you know about antibiotic resistance? |  |
| 315 | From your perspectives, what do you think would be the consequences or bad effects of the use of antibiotics? |  |
